# High-level context effects on spatial displacement: the effects of body orientation and language on memory

**DOI:** 10.3389/fpsyg.2014.00637

**Published:** 2014-07-03

**Authors:** David W. Vinson, Drew H. Abney, Rick Dale, Teenie Matlock

**Affiliations:** Cognitive and Information Sciences, School of Social Sciences, Humanities and Arts, University of CaliforniaMerced, CA, USA

**Keywords:** representational momentum, motion simulation, spatial displacement, motion comprehension, language comprehension, body orientation

## Abstract

Three decades of research suggests that cognitive simulation of motion is involved in the comprehension of object location, bodily configuration, and linguistic meaning. For example, the remembered location of an object associated with actual or implied motion is typically displaced in the direction of motion. In this paper, two experiments explore context effects in spatial displacement. They provide a novel approach to estimating the remembered location of an implied motion image by employing a cursor-positioning task. Both experiments examine how the remembered spatial location of a person is influenced by subtle differences in implied motion, specifically, by shifting the orientation of the person’s body to face upward or downward, and by pairing the image with motion language that differed on intentionality, *fell* versus *jumped*. The results of Experiment 1, a survey-based experiment, suggest that language and body orientation influenced vertical spatial displacement. Results of Experiment 2, a task that used Adobe Flash and Amazon Mechanical Turk, showed consistent effects of body orientation on vertical spatial displacement but no effect of language. Our findings are in line with previous work on spatial displacement that uses a cursor-positioning task with implied motion stimuli. We discuss how different ways of simulating motion can influence spatial memory.

## INTRODUCTION

**FIGURE 1:**
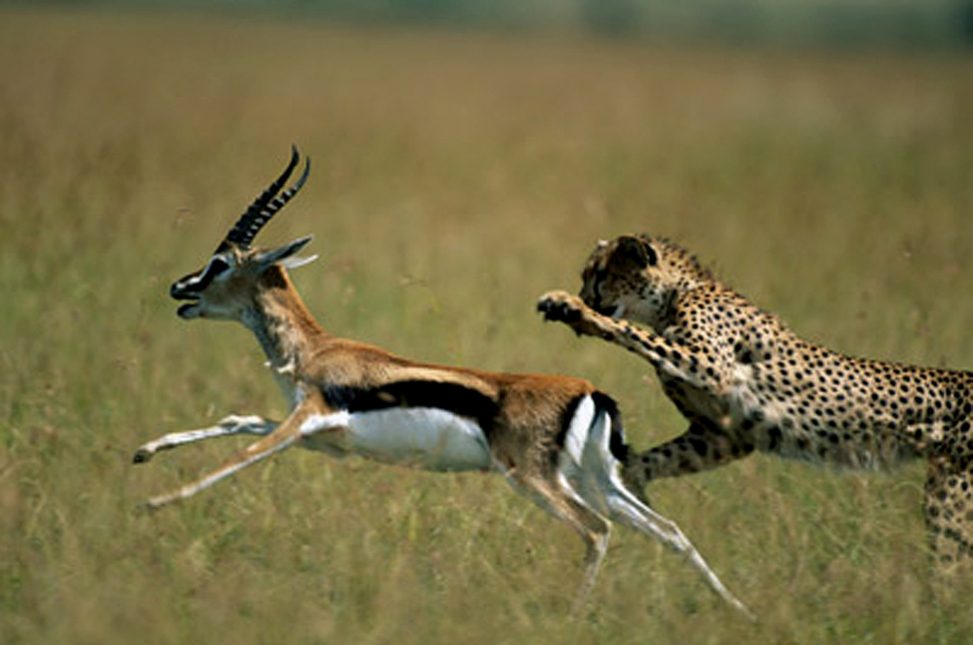
**An example of implied leftward motion**.

Observers often report that the position of a static or frozen-action object appears to be displaced in the direction of implied motion. For example, in **Figure 1** the cheetah is chasing a gazelle, and the direction of implied motion of the cheetah is leftward.

When people view static action images, such as cheetah chasing the gazelle in **Figure 1**, motor areas associated with the perception of actual movement are actively engaged (Kourtzi and Kanwisher, 2000), suggesting that simply viewing implied motion can lead to perceptual motor simulations of movement. This is further supported by the observed perceptual effects that arise while viewing frozen-action images that imply motion. Specifically, when asked later to indicate the position of a previously observed object that displays actual or implied motion (e.g., the cheetah) its remembered location is typically displaced in the direction of motion (Freyd, 1983; Freyd and Finke, 1984). There is some debate about what such spatial displacement effects suggest about brain activity. On the representational momentum view (e.g., Freyd, 1987), people are thought to simulate future motion, and in doing so, displace a moving object slightly farther along its path of movement. On another view, however, it is believed that object displacement can be accounted for by the smooth pursuit of the eyes tracking a moving stimulus (Kerzel, 2003). Still, some amount of displacement cannot be accounted for by objective behavioral measures such as gaze pursuit alone (see Hubbard, 2005, for review). Indeed motion simulation theories have been used to account for many other effects across many areas of cognitive science. Some researchers posit the occurrence of motion simulation as a way to comprehend the perception of motion in others (Blake and Shiffrar, 2007) as well as motion implied in language (Glenberg and Kaschak, 2002; Matlock, 2004, 2010; Casasanto, 2009; see Glenberg et al., 2013, for review). For example, in Loftus and Palmer (1974), participants watched a car accident video and later answered a question about how fast the cars were going when they smashed into each other. In some cases, the prompt featured another motion verb, for instance, *bumped* or *hit*. When the verb *smashed* was used, participants reported the car had been going faster (versus other motion verbs). The participants also inaccurately reported that there was broken glass in the accident, even though there was none. If motion simulation is ubiquitous across different cognitive domains, it may be that the contextual constraints that characterize some aspects of an object’s motion, such as its orientation or how it is described, may affect how it is perceived.

Recent neurological evidence supports the idea that motion simulation is involved in action observation and language comprehension. Studies using fMRI have shown greater cortical activation in areas associated with motor action when expert dancers view other experts of their own dance (e.g., Ballet) compared to when they view experts of another dance (e.g., Capoeira; Calvo-Merino et al., 2005). Indeed, brain areas closely associated with motion comprehension become active in the perception of object motion and comprehension of implied motion in language. Brain regions V5/MT in particular are activated when viewing frozen-action images (Senior et al., 2000). Activation is mediated by an image’s preceding linguistic context. For example, a still image of a box of noodles pouring over a pot is associated with more motion processing activation when preceded by a sentence with a spatial relation (“the box is over the pot”) than a sentence with a comparative adjective (“the box is bigger than the pot” Coventry et al., 2013). When these areas are inhibited via Transcranial Magnetic Stimulation, over 60% of all participants show no stereotypical spatial displacement effects (Senior et al., 2002). Given this, the position a cheetah that was said to have “darted forward” or “leaped forward” when facing one direction or another might be remembered differently. Such a finding would provide new insights into how motion simulation in comprehending language and observing action affect spatial memory.

The purpose of the current study was to examine how differences in these domains might affect spatial memory for objects in a scene. We examine how the remembered spatial location of an agent is affected by manipulations of the current visual and linguistic context. Considering the effects found in previous research, our hypothesis is that visual and linguistic information may act as contextual constraints influencing the remembered location of an agent. Before presenting results of the current study, we briefly review research on motion simulation. This review motivates the idea that contextual parameters might lead to differences in motion understanding including the comprehension of bodily orientation and motion language.

## MOTION SIMULATION

People simulate future motion based on current motion observed in the world. Signs of motion simulation occur early in development. When infants observe a toy car role behind an occluded space, they are able to infer whether or not the car will have a safe passage to the other end of the space—whether there is a block in the way of the car’s trajectory behind the occlusion or not (Baillargeon, 1986). Differences in motion simulation revealed through differences in the remembered location of actual or implied motion objects are dependent on many physical variables that influence spatial memory (see Hubbard, 2005, for review). One robust physical variable shown to impact the remembered location of objects in space, and crucial to the current study, is gravity. When participants observe a man who seemingly hopped off a curb, memory for the man’s position is displaced in the direction of gravity (Freyd, 1983). It may be that motion simulations incorporate constraints imposed by our environment (Shepard, 1984), one being gravity (Hubbard, 1990). Additionally, Hubbard (1994) suggests the effects of motion simulation may be strongest along the path the target is believed to travel (see also Hubbard, 2006). Other factors include stimulus velocity (Hubbard and Bharucha, 1988), visually tracking the stimulus (Kerzel, 2003), stimulus control and observation (Jordan and Hunsinger, 2008), local context (Hubbard, 1993), and conceptual understanding (Reed and Vinson, 1996).

Effects of motion simulation are not limited to spatial variables. One recent study showed that increasing the time between object observation and recall lead to increased spatial displacement in the direction of simulating gravitational forces (De Sá Teixeira et al., 2013). When time between the presentation of a horizontally moving stimulus and stimulus placement exceeded 300 ms, spatial displacement occurred in a vertical direction. Vertical displacement increased as lag time increased to 1000 ms, indicating that lag time might be occupied by gravitational motion simulation. This further suggests that simulating the future motion of an object occurs over time and space. The visual system can differentiate between highly specified motions, such as those of an agent, which might have interesting effects on object memory. In particular, the visual system is sensitive to human bodily motion (Shiffrar and Thomas, 2012). The direction of motion of the body, for instance, can be identified from impoverished stimuli (e.g., point light walkers) nested within a noisy scene (Bertenthal and Pinto, 1994). Further, the visual system can differentiate human body movement from basic object movement (Shiffrar et al., 1997) and animal movement (Cohen et al., 2002). The visual system’s sensitivity to specific features can influence actions as well. Expert rugby players are more likely to anticipate deceptive movements of an opponent and act on honest movements compared to novices when observing the kinematics of their opponent’s movements (Brault et al., 2012; see also Mori and Shimada, 2013). More generally, this suggests perceptual differences in the orientation of one’s body influence observer actions.

Neural activation during action observation indicates the visual system’s sensitivity to the human body may result from the spontaneous activation of motor cortical areas associated with one’s ability to act (Blakemore and Decety, 2001; see also Ambrosini et al., 2012). This provides insights into how the actions and intentions of another person can be predicted merely by observations of bodily movement (Sebanz and Shiffrar, 2009), and why viewing another person’s movements while dancing activates motor cortical areas differently in experts and in novices who are not currently dancing (Calvo-Merino et al., 2005). Collectively, the ability to comprehend or perceive specific actions may be tightly coupled to one’s own ability to act and plan similar actions (Buccino et al., 2001; Hommel et al., 2001; Rizzolatti et al., 2001). As a result, the simulation of specific human movements may be important to the perception and memory of other’s movements in relation to our own. If so, observed differences in body orientation should lead to different action simulations that are consistent with observed orientations.

Linguistic information about orientation can influence visual observation as well. Stanfield and Zwaan (2001) discovered that participants were faster to respond to the visual presentation of an object when its orientation was congruent with prior linguistic descriptions about orientation than when image orientation and language were incongruent. This finding was taken as evidence that comprehension involves the perceptual simulation of the object’s position in space (Barsalou, 1999). Indeed, simulating the properties of objects specified by linguistic information is important for comprehension (Glenberg, 1997; Zwaan and Radvansky, 1998; Glenberg et al., 2010). For example, when movement is similar to the implied movement of a statement such as *close the drawer*, participants are quicker to judge the direction of motion (Glenberg and Kaschak, 2002). Evidence for simulation in language is also shown across a variety of other empirical work (see Barsalou, 2003; Barsalou et al., 2008; Zwaan and Madden, 2005). Further support for motion simulation in language comprehension is shown in research on motion verbs (Loftus and Palmer, 1974), fictive motion sentences (Matlock, 2004; Matlock et al., 2005; Richardson and Matlock, 2007), and grammatical aspect (Madden and Zwaan, 2003; Anderson et al., 2010; Bergen and Wheeler, 2010; Huette et al., 2012; Matlock et al., 2012). This constellation of work suggests that the effects of motion simulation on spatial memory are influenced by various linguistic properties.

Given that basic motion simulation is supported by findings from studies on spatial displacement, it is plausible that other domain-specific factors that are thought to invoke motion simulation, including those specific to comprehending motion in the human body and language, may also influence spatial memory. Specifically, different bodily orientations and different motion verbs such as *fell* or *jumped* might have an impact on how the location of an agent in space is remembered.

## METHODS IN REPRESENTATIONAL MOMENTUM

Two methods are often used to investigate the effects of motion on spatial memory: A probe-judgment task is used in implied motion tasks and a cursor-positioning task used in actual motion tasks (see Hubbard, 2005, for review). A probe-judgment task is typically used for static images that imply motion. In these studies, a single static image is shown multiple times in slightly different positions, such as in a clockwise motion (Freyd and Finke, 1984). This is followed by a probe image, with the same image either farther along in the direction of implied motion or in the same location as the previous image. Subjects are instructed to determine if the probe image is the same or different from the last test image. The probe-judgment task has also been used to assess the impact of frozen-action images on spatial memory (Freyd, 1987). In one study, participants were presented with a test stimulus, a frozen-action image often cut from a video, and then a second frozen-action probe image 250ms later. In this case the probe image is a scene from the same video occurring moments before or moments after the first image (Freyd, 1983). When the second image is congruent with continued motion, participants have a harder time indicating that the first and second images are different. Freyd’s results suggest frozen-action images influence spatial memory when the implied motion of the probe stimulus is congruent with actual or possible motion. However, it is difficult to determine if these findings disentangle whether the effects of implied motion are the result of the single presentation of frozen-action images or the presentation of both the test image and the probe image presented sequentially. To be sure, the use of a probe-judgment task can only indirectly assess the exact impact of implied motion that stems from the presentation of a single initial image.

The use of a frozen-action probe can control for differences in judgment that might occur from indicating the position of a stimulus with, for example, a cursor. The physical action of moving one’s arm using a cursor-positioning task may introduce task demands that can affect the indicated placement of a stimulus. For example, if one were to move the cursor from the bottom of the screen to indicate the location of a missing object, it would be difficult to ascertain whether spatial displacement in the direction of implied gravity is the result of simulating gravity or of task demands from moving one’s own arm. Typically, cursor-positioning tasks are used for actual motion, but probe-judgment tasks have also been used (see Kerzel, 2003). In cursor-positioning tasks, after a moving stimulus has vanished, participants place the cursor on the remembered vanishing point of the stimulus. This measure directly assesses one’s memory of the location of a stimulus.

Cursor-positioning tasks are not usually used to test the effects of a single frozen-action image on spatial memory. In part, this is due to controlling for participant movements that might affect stimulus placement. Alternatively, it is possible that a cursor-positioning task used in the presentation of a frozen-action image could disentangle whether the effects of implied motion stem from apparent motion (see Kolers, 1972) or from the simulation of a single frozen-action image. This method would also allow for constraints imposed on a frozen-action image such as the orientation of the image or language specifying image motion to be more directly assessed.

### THE PRESENT RESEARCH

**FIGURE 2:**
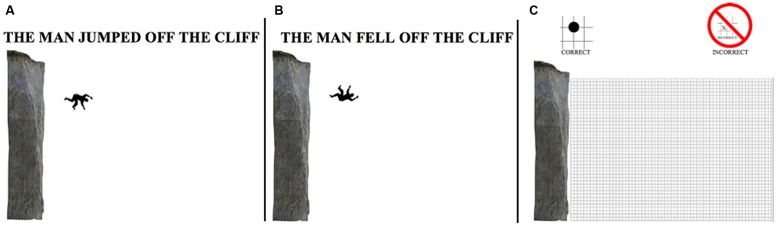
**Example condition.** Left: **(A)** page one; condition jumped/down. Center: **(B)** page one; condition jumped/up. Right: **(C)** Page two; response page with cliff and Cartesian grid.

The main goal of the current study is to investigate if the effects of implied motion from frozen-action images, seen in previous studies, can be observed using a cursor-positioning task. We explored how specific differences in implied motion, precisely, differences in body orientation and language, affected memory for the location of a man’s body. In two experiments, participants viewed an image of a cliff and a silhouette image of a man’s body to the right and below the cliff’s edge implying a descending gravitational motion. We examined the effects of body orientation (either facing upwards or facing downwards) and specific motion verbs (either *fell* or *jumped*) on memory of the man’s body (**Figure 2**). In doing so, we assessed how other factors influencing motion simulation collectively affect spatial memory. Experiment 1 examined how these differences might influence spatial memory using a survey methodology. Experiment 2 was designed to replicate the effects of Experiment 1 using a computer-based program and a different population: a large sample recruited from Amazon’s Mechanical Turk.

## EXPERIMENT 1

We used a cursor-positioning method to examine how spatial memory is affected by implied gravity, body orientation of an implied motion figure, and language. We presented participants with a frozen-action image of a man next to a cliff oriented to be facing up or down in addition to a description of his actions. The spatial location of frozen-action image presented in a congruent sequence (e.g., direction of implied gravity) is typically remembered as more displaced than when presented within an incongruent sequence (Freyd and Finke, 1984). Using a single frozen-action image, in contrast to a series of images, might reveal the influence of implied gravitational forces on spatial memory that stem from the observation of a single image. For the current experiment, differences in anticipated or simulated motion of the observed man may occur with different body orientations. Specifically, the kinematics of the body suggest that having the capacity to push off or away from a cliff may be more probable when facing down than when facing up. As for language, simulated differences may occur between motion verbs such as *jumped* and *fell*. Having jumped may indicate a different trajectory in spatial memory than having fell.

### METHOD

#### Participants

A total of 305 (*M*_age_ = 18.38, SD_age_ = 1.44) undergraduate students from the University of California, Merced, participated for extra course credit. Data from 16 participants were not analyzed because they failed to follow instructions (e.g., placed a dot in between crosshairs, making it impossible to determine the exact *x, y* coordinates intended by the participant) (only about ∼5% of the entire data set). This is further elucidated in procedures below.

#### Materials and design

All participants viewed an 8.5″ × 11″ black and white picture of a cliff and a silhouette figure of a man (see **Figure 2A**). Having scaled the height of cliff based on the typical height of a human male (1.8 m) the relative size of the cliff was 10.26 m. The man’s orientation was either facing upward (Up) or facing downward (Down). His orientation was ambiguous: He could appear to have fallen off or to have jumped off the cliff. In both conditions, the center of the man’s body was located at the same *x, y* coordinates (2 m, -2 m) when the origin was located at the top of the cliff edge. This is approximately 2 m away from the cliff and 2 m below the cliff edge. Given the man’s vertical distance from the cliff (2 m) and gravity (9.8 m/s^2^) the length of time the man appears to have been falling can be calculated by using his vertical distance (*d* = 2 m): *t* = sqrt(2*d/G) = 0.63 s. From this, the horizontal velocity of the man can be calculated using the horizontal distance form the cliff (*d* = 2 m): *d/t* = 3.13 m/s. Additionally, the average velocity, calculated using the distance from the edge of the cliff to the man (*d* = 2.82 m) was 4.43 m/s.

For the language manipulation, one of three statements appeared just above the image: (1) “THE MAN FELL OFF THE CLIFF”, (2) “THE MAN JUMPED OFF THE CLIFF”, or (3) nothing (control). The experiment was a 3 (language: *Fell* vs. *Jumped* vs. No language) × 2 (orientation: “up” vs. “down”) between-subjects design. Each subject was randomly assigned to 1 of 6 conditions: fell/up, fell/down, jumped/up, jumped/down, no language/up, no language/down. On page two of the survey, all participants observed the cliff from page one again, but this time with no language and with no man. The image of the cliff and the addition of a Cartesian grid overlay were included (see **Figure 2B**). There were 46 × 63 cells, with each cell corresponding to an area of 0.22 m^2^. The dimension of the grid overlay was 10.26 m × 13.86 m (*height*× *width*). Henceforth, all coordinates are listed in meters relative to the estimated size of the man.

#### Procedure

Participants completed a packet of various survey-based tasks, including the current experiment. Every participant observed the experiment in the same order on the same page in the packet. The experiment was not placed adjacent or near other experiments that related to space or motion.

Participants were instructed to study the first page for 10 s, turn over to page two, and answer questions about the image previously viewed on page one. On page one, participants read, “*NOTE: YOU ARE NOT ALLOWED to return to this page when answering questions on the next page*”. The instructions at the top of page two included: “*WITHOUT RETURNING TO THE PREVIOUS PAGE using the grid below, place 1 DOT = the location of the CENTER of the man.*” An image presented below the instructions indicated that participants were to place the dot on a crosshair of the grid (see the top of **Figure 2C**). Using the cliff as a reference point, participants were to place a large dot on an *x, y* coordinate of the Cartesian grid next to the cliff at the remembered location of the man. The *x, y* coordinate from each participant’s perceived estimate was recorded.

### RESULTS

Differences between the remembered man and the actual man collapsed across all conditions were analyzed, followed by differences between the remembered man dependent on both orientation and language conditions. To examine how orientation and language would influence the placement of the man, both *x*- and *y*-coordinates were assessed. All results are presented in meters to provide a real world metric of the remembered position of the man.

#### Remembered location

To determine if the remembered location of the man was significantly different from the actual location of the man, *t*-tests were performed to determine the overall differences between *x*- and *y*-coordinates of the man’s location from the cliff edge. *T*-tests are permitted because each datum represents a unique participant and can be assumed to be independent from the other observations.

For the *x*-coordinate, the remembered location (*M* = 2.14 m, SD = 0.66 m) was placed reliably farther to the right of the actual location (μ = 2 m), *t*(362) = 4.66, *p* = 0.001. For the *y*-coordinate, the remembered location (*M* = -3.12 m, SD = 0.91 m) was reliably lower than the actual location (μ = -2 m), *t*(362) = -14.64, *p* = 0.001.

**FIGURE 3:**
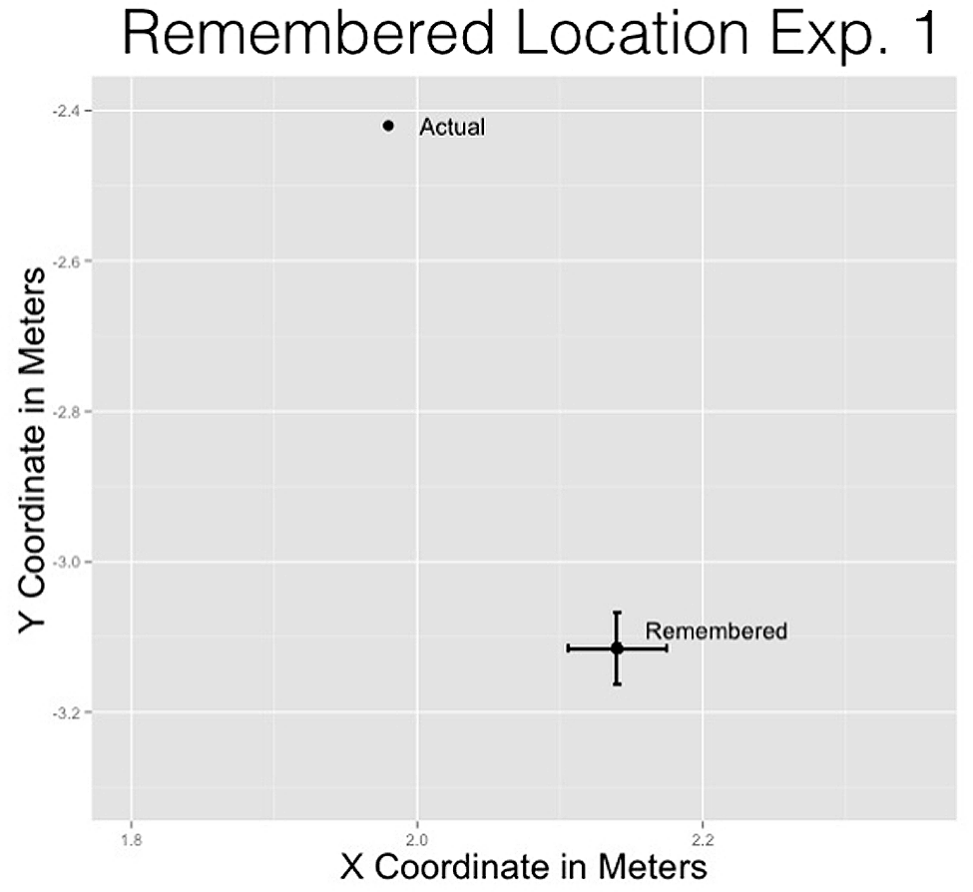
**Remembered location of the man compared to the actual location of the man for Experiment 1**.

The *x*- and *y*-coordinate results provide evidence consistent with previous representational momentum results suggesting that the man was remembered displaced from his actual position in the direction imposed by gravitational forces (see **Figure 3**).

#### Orientation and language

A 3 (Language: “Fell” vs. “Jumped” vs. No language) × 2 (Orientation: Up vs. Down) between-subjects ANOVA was performed on *x*-coordinates and *y*-coordinates separately. We report all significant results below. No significant main effects or interactions were observed for the *y*-coordinate.

**FIGURE 4:**
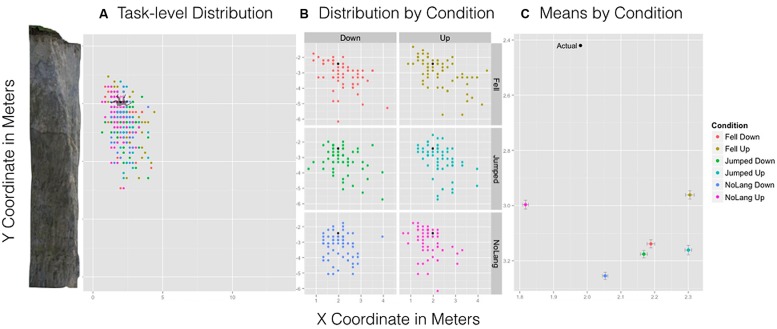
**Experiment 1 results.** Left: **(A)** A task-level view of the distribution of responses for all six conditions with the cliff and man scaled to size. Center: **(B)** The spread and density of responses broken down by condition with point (black) indicating the actual position of the man. Right: **(C)** The mean estimated location of the man by condition and standard error bars for *x*- and *y*-axes.

We hypothesized that differences in the type of language and orientation would influence the remembered spatial location of the man relative to the cliff. There was a significant Language × Orientation interaction for the *x*-coordinate, *F*(2,357) = 3.22, *p* = 0.04 (see **Figure 4C**). This effect was driven primarily by the no language/up condition. Participants remembered the location of the man in the no language/up condition to be significantly closer to the cliff than all other conditions. Pairwise comparisons with Bonferroni–Holm correction indicated that the interaction was influenced by this condition: No language/up condition (*M* = 1.82 m, SD = 0.51 m) was more leftward, or closer to the cliff, relative to No language/Down (*M* = 2.05 m, SD = 0.58 m, *p* = 0.01), Fell/Down (*M* = 2.19 m, SD = 0.68 m, *p* = 0.02), Fell/Up (*M* = 2.31 m, SD = 0.82 m, *p* = 0.001), Jumped/Up (*M* = 2.30 m, SD = 0.57, *p* = 0.001), and Jumped/Down (*M* = 2.17 m, SD = 0.64 m, *p* = 0.03). **Figure 4A** shows the actual position and size of the man relative to the cliff overlain by the spread and density of participant responses for all conditions. **Figure 4B** shows the spread and density of estimates by condition showing a marked difference between no language/up and all other conditions. Importantly, this interaction suggests that the presence of language influenced one’s memory for implied motion images. Note that in this condition the remembered location of the man was closer to the cliff than the actual location of the man.

### DISCUSSION

Representational momentum influenced where participants placed the man relative to the cliff. Orientation and language also influenced the placement of the man. Understanding the constraints of the task can further elucidate this interaction.

Both *x*- and *y*-coordinates indicated the remembered location of the man to be different than the actual location of the man. This finding is in line with the implied effects of gravity found in previous spatial displacement studies, but the task involved a cursor-positioning task with a frozen-action image. Using a cursor-positioning task allowed for control of potential implied motion effects that could have been due to apparent motion occurring from presenting the stimulus more than once, as in the case of a probe judgment task (Freyd, 1987). Crucially, in this condition the remembered location of the man was *behind* his actual location (i.e., close to the cliff). This suggests that if participants are simulating motion, it may not be that of the man’s specific trajectory. Therefore, the results from Experiment 1 suggest that the remembered location of a static object in space can be said to involve the simulation of gravitational motion in general.

The interaction of language and orientation on spatial memory observed in this experiment can be explained by considering the constraints of the task. There was a significant interaction such that when no language was present and the man was facing upward, the remembered location of the man was reliably closer to the cliff than in any other condition. It is possible this resulted from how language was presented in contrast to its implied motion contents. The presentation of language at the top of the scene may have pulled the remembered location of the image farther out, with respect to the left-to-right eye movements during reading (Rayner and Schotter, 2013). This is supported by findings showing that where the eyes are located when observing a stimulus influences the remembered location of that stimulus (Kerzel, 2000; Kerzel et al., 2001).

It is possible that an effect of orientation on the horizontal plane exists such that “Up” is significantly closer to the cliff than “Down,” but masked by how language was presented in this experiment. This effect would support a simulation account of motion that is more sensitive to the body, though not necessarily language. We speculate this difference would suggest the simulation of bodily motion is different than the simulation of more basic gravitational motion. Such subtle influences on spatial memory suggest motion specific to the body may involve the simulation of future bodily positions implied by current bodily positions. This hypothesis is further explored in Experiment 2.

## EXPERIMENT 2

The goal of Experiment 2 was twofold: to acquire more accurate participant responses and to test the effect of language when presented *before* (rather than *with*) the scene. This was possible with an online experiment that used Adobe Flash CS6. In this experiment, participants were unable to return to previous scenes, or stay on the same scene for more than a few seconds. This provided greater control over the presentation of language and participant responses.

The presentation of the cliff and man was the same as it was in Experiment 1. However, to control for potential influences of eye movements on spatial memory, linguistic information was presented prior to the image (in Experiment 1 participants viewed the image and sentence on the same page at the same time). Another important difference between experiments involved how sensitive the task was to participant responses. Specifically, in Experiment 1, participants were given the task of providing an estimate within the constraint of a Cartesian grid. This reduced the number of estimates in the *x, y* coordinate space of 2898 (46 × 63) possible locations. To increase the number of possible estimate locations, the program created and used for Experiment 2 afforded 169,371 (369 × 459) possible *x, y* coordinate locations. Increased sensitivity in this experiment should provide a more accurate measure of participant responses. The use of a visible grid during estimation was not necessary given the ability to extract exact coordinates directly from the program and thus was not used in Experiment 2.

### METHOD

#### Participants

One thousand and eight Amazon Mechanical Turk users from the United States participated in exchange for $0.10 USD. All participants were required to have an updated version of the Adobe Flash player. After choosing to complete the task, participants were directed to a web-link containing an interactive Adobe Flash CS6 program^1^. As reported below, despite much larger power, language had no obvious impact on spatial memory. Data from 36 participants were excluded from the analysis because responses were three standard deviations away from either the mean of *x*- or *y*-coordinate responses (∼3.5%).

#### Materials, design, and procedure

**FIGURE 5:**
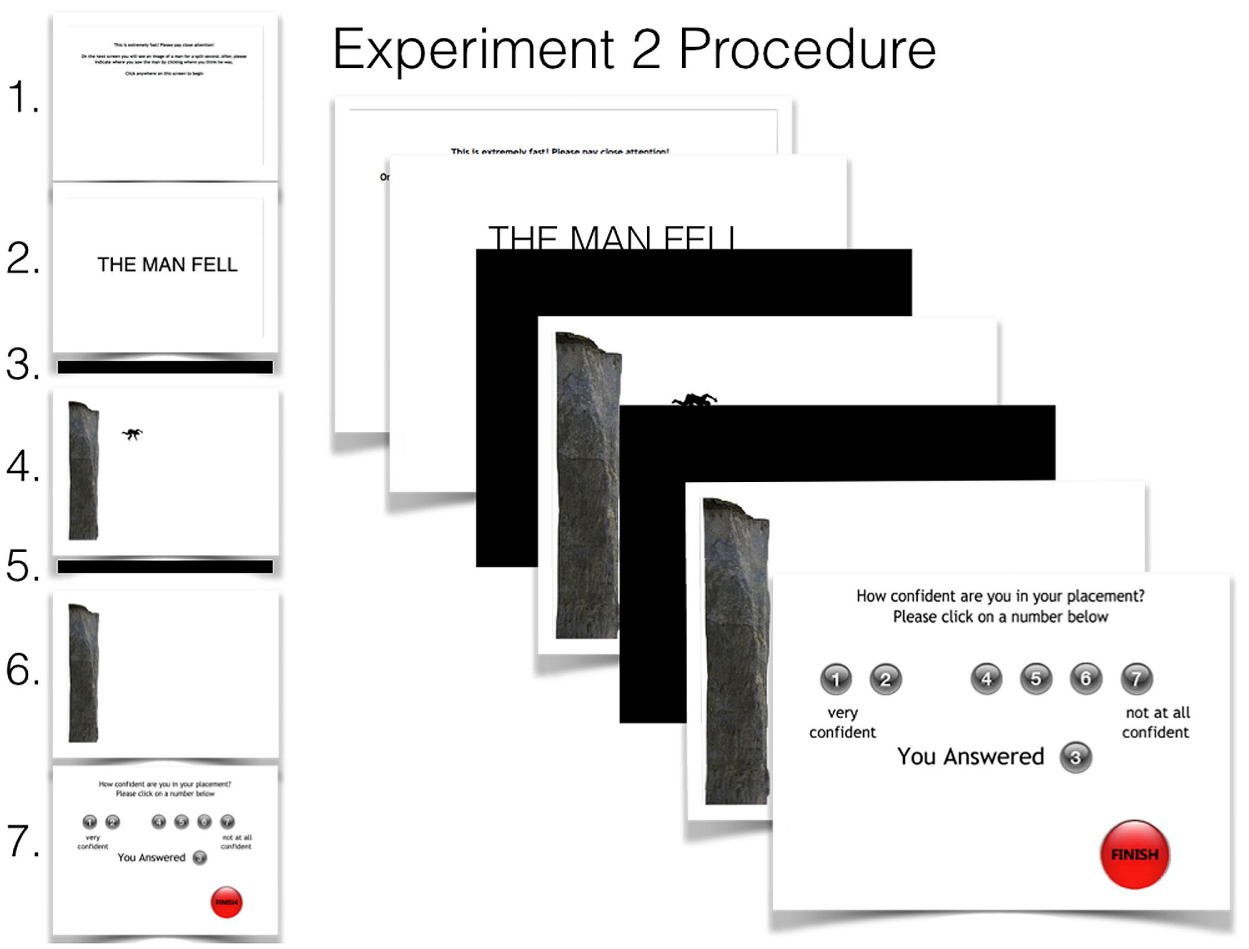
**Experiment 2 procedure.** Participants observed each scene (left) in sequential order from top to bottom starting with (1) instructions, (2) test sentence, (3) black screen mask, (4) test image, (5) black screen mask, (6) response screen, then (7) the confidence measure. During the presentation of the response scene only the participant’s cursor appeared as the image of the man. The final scene debriefing participants is not shown.

An Adobe Flash CS6 program was used to design this experiment. Participants observed a total of 8 screens, each with content that differed from the previous scene (see **Figure 5**). Because each scene was fit to the participant’s computer screen exact pixel dimensions are unknown. However, all scenes were presented in Adobe Flash stage *height* × *width* dimensions (400 × 550 pixels), so the relative dimensions were used. In Adobe Flash, the origin of the screen is located at the top-left corner of the screen meaning all possible x-coordinates are positive while all *y*-coordinates are expressed as negative values. For the purpose of this experiment, and consistent with Experiment 1, we considered the point of origin as the top of the cliff’s edge. This results in a space of 369 × 459 possible *x, y* points. Further, the upper-left corner of each object was used as the object’s reference point to the scene. Thus, the *x, y* coordinate of all objects describe the position of the upper-left corner of each object, respectively.

In the experimental software, the first screen displayed these instructions in black font on a white background: “*This is extremely fast! Please pay close attention! On the next screen you will see an image of a man for a split second. After, please indicate where you saw the man by clicking where you think he was. Click anywhere on this screen to begin”.* After clicking on the screen, the participant’s cursor disappeared and screen two appeared for 2000 ms. Screen two contained a white background with one of the same three language conditions used in Experiment 1. A single text box (200 × 550) with no border contained the test sentence (font: Trebuchet, bold; size: 25) presented in the center of the screen with the top left corner of the box located at (0–100). This was automatically followed by screen three, a black backdrop mask for a 1000 ms duration. Screen four was presented for 1000 ms and contained the test image of the cliff (400 × 91) and the man (35 × 60) that was presented in Experiment 1. The cliff’s edge (369 × 91) closest to the man was considered the origin (0, 0) and the same image of the man (35 × 60) was positioned at coordinate (59, -49) relative to the cliff edge. The relative height of the cliff given the average height of a man (1.8 m), from base to origin, was 11.07 m. From this, the position of the man from the cliff was displaced 1.77 m horizontally from the cliff and -1.47 m vertically from the cliff’s edge. Each possible *x, y* coordinate contained an area of 0.03 m^2^. Given the man’s vertical displacement from the cliff’s edge was shorter than that in Experiment one, the amount of time he appears to have been falling from the cliff was slightly shorter; approximately 0.54 s. From this, the man’s horizontal velocity was determined to be 3.22 m/s. Additionally, the average velocity, calculated using the distance from the edge of the cliff to the man (*d* = 2.3 m) was 4.25 m/s. The size of the cliff and placement of the man was not intended to replicate that of Experiment 1, but to represent some gravitational and horizontal velocity more generally. Image orientation was identical to the orientation used in Experiment 1. Screen five contained a black backdrop mask identical to screen three for 1000 ms. This replicated previous presentation and response masking times used in recent studies investigating the temporal effects of spatial memory (De Sá Teixeira et al., 2013).

This was followed by screen six, which was identical to screen four with the exception of the man omitted from the scene. The cursor, having disappeared at the presentation of screen two, now re-appeared but this time, instead of a mouse, it was the exact image of the test stimulus (e.g., the man) seen on screen four, oriented exactly the same. Participants were instructed to click on the screen by dragging the man to where they remembered the man to have been, as indicated by previous instructions^2^. After an estimate was made, screen seven appeared with a confidence rating scale. Instructions at the top read: “*How confident do you feel in your placement? Please click on a number below*.” Below the number 1 read “*very confident*” and below the number 7 read “*not at all confident*”. Once participants had completed the confidence rating, they were instructed to click on a “*FINISH*” button in the bottom right corner of the screen.

### RESULTS

#### Remembered location

*T*-tests were performed to determine if the remembered location of the man would reliably differ from the original position of the man. These tests assessed overall differences among *x*- and *y*-coordinates of the remembered location of the man and the actual location of the man from the cliff edge. All results are presented in meters.

**FIGURE 6:**
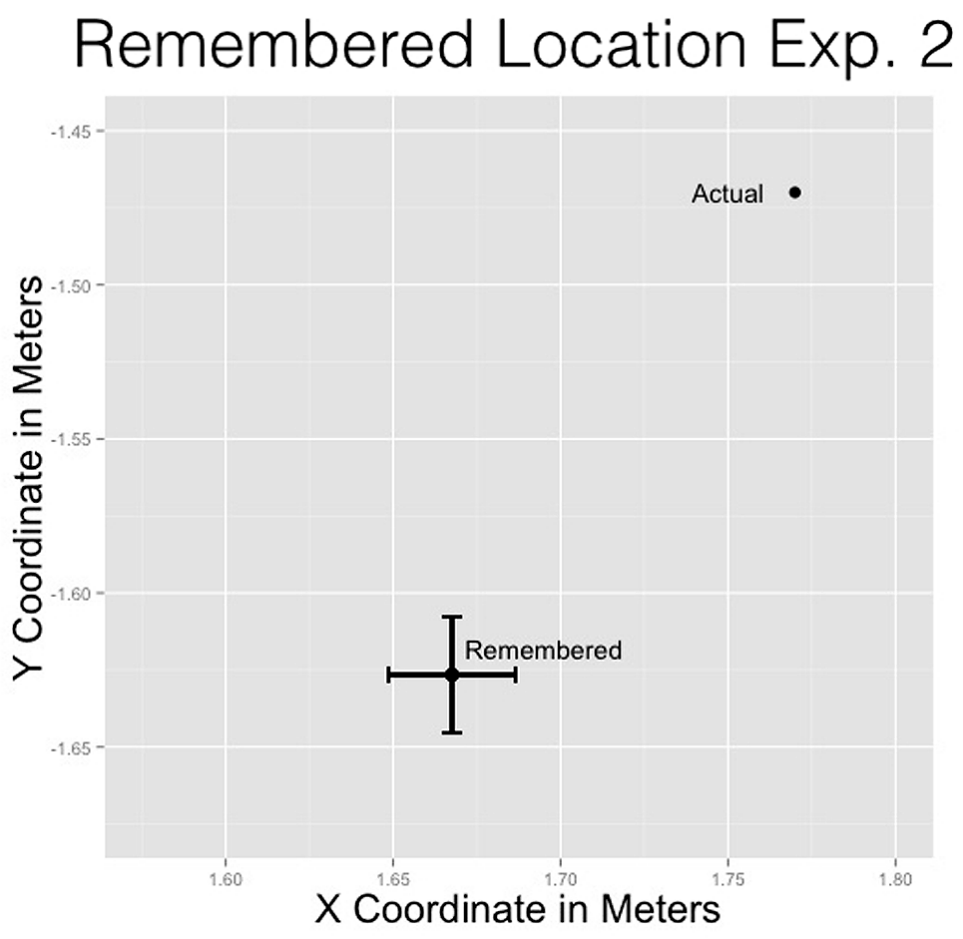
**Remembered location of the man compared to the actual location of the man for Experiment 2**.

For the *x*-coordinate, the remembered location (*M* = 1.67 m, SD = 0.59 m) was closer to the cliff than the actual location (μ = 1.77 m), *t*(972) = -5.42, *p* < 0.001. This replicates previous effects showing the remembered location of a moving stimulus to be displaced opposite the direction of motion and downward (De Sá Teixeira et al., 2013). For the *y*-coordinate, the remembered location (*M* = -1.63 m, SD = 0.58 m) was significantly lower than the actual location (μ = -1.47 m), *t*(972) = 8.37, *p* < 0.001 (see **Figure 6**).

#### Orientation and language

Consistent with Experiment 1, a 3 (Language: “Fell” vs. “Jumped” vs. No language) × 2 (Orientation: Up vs. Down) between-subjects ANOVA was performed on *x*- and *y*-coordinates separately. We report all significant results below. Consistent with Experiment 1, no significant main effects or interactions were observed for *y*-coordinates.

**FIGURE 7:**
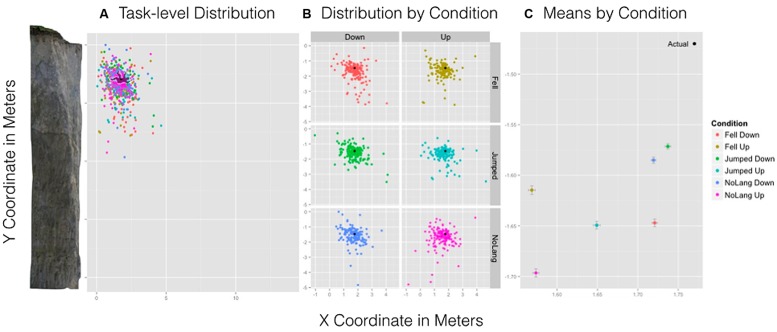
**Experiment 2 results.** Left: **(A)** A task-level view of the distribution of responses for all six conditions with the cliff and man scaled to size. Center: **(B)** The spread and density of responses broken down by condition with point (black) indicating the actual position of the man. Right: **(C)** The mean estimated location of the man by condition and standard error bars for *x*- and *y*-axes.

For the *x*-coordinate, a significant main effect of Orientation suggested that Up (*M* = 1.60 m, SD = 0.60 m) was remembered to be closer to the cliff relative to the Down condition (*M* = 1.73 m, SD = 0.58 m), *F*(1,697) = 11.52, *p* < 9.001 (see **Figure 7C**). **Figure 7A** shows the actual position and size of the man relative to the cliff overlain by the spread and density of participant responses for all conditions. **Figure 7B** shows the distribution of location estimates for all conditions. All other main effects and interactions were not significant.

#### Confidence

No effect of confidence for *x*- or *y*-coordinate or interactions among confidence, language, and orientation were observed.

### DISCUSSION

In Experiment 2, the man was remembered to be farther along a gravitational trajectory than his actual position, which is consistent with Experiment 1 and with previous findings (e.g., Hubbard, 2005). Experiment 2 showed the same implied gravitational effects in Experiment 1 but provided greater accuracy and control over response measures.

The procedure for Experiment 2 allowed participants to respond by using the same image of the man seen in the experimental scene. This eliminated potential limb location effects of orientation. Importantly, observing that the remembered location of the man while oriented upward was significantly closer to the cliff than when oriented downward suggests that the interaction obtained in Experiment 1 may have resulted from task demands. In other words, the mere presentation of language influenced the remembered spatial location of the man. This suggests that the orientation differences in Experiment 2 replicate the simple effects of Experiment 1 when no language was presented^3^.

The difference in both language conditions for the *x*-coordinate measure compared to No language was not replicated in Experiment 2. In Experiment 2, language was presented before the image, but in Experiment 1, language and image were presented simultaneously. The method of presentation order of linguistic information may be one reason why there was an effect of language on the remembered location of the image in Experiment 1 but not in Experiment 2. This is supported by previous studies showing that where the eyes fixate influences an object’s remembered location (Kerzel, 2000; Kerzel et al., 2001). The effect of language in Experiment 1 was not replicated in Experiment 2 and is most likely not the result of linguistic content. Crucially, the placement of the man was behind the man’s actual position replicating the results of Experiment 1 when no language was presented and the man was facing up. This again suggests that exact trajectories implied by both cliff and man may not be directly simulated; though specific simulations of gravity may be affected by the observed kinematics implied by the current posture or position of a human body.

## GENERAL DISCUSSION

Memory for where an object is located in space can be influenced by actual or implied motion associated with that object (Hubbard, 2005). The results of this work are consistent with this well-known finding. Previous research shows basic motions, such as gravity and horizontal movement, can influence spatial memory (Hubbard, 2005; De Sá Teixeira et al., 2013). It is possible that the effects we observed are the result of simulated motion, or of predicting the outcome of observed motion (Jordan, 2009). The most robust finding in our experiments is the significant displacement of the man in the direction of gravity. Crucially, the displacement of the man was closer to the cliff than actual. This suggests that a specific trajectory—one of a falling object—might not be simulated. Instead, this effect may be a characteristic more closely related to the observation of actual or implied motion stimuli, in general, over longer time scales. This finds support from a recent study by De Sá Teixeira et al. (2013) showing the remembered location of a horizontally moving stimulus is displaced vertically (below) and behind the stimuli’s actual position when response time was delayed for 1000 ms. This was compared to response time delays of less than 1000 ms that show no backward horizontal displacement. It is possible that with a delay between presentation and response of less than 1000 ms, the backward horizontal displacement might not have occurred, showing instead a more accurate simulation of a falling body’s trajectory. Understanding the mechanisms that underlie this curious effect will require further investigation.

Though a shorter delay may have brought on a more accurate simulation of the implied horizontal movement of a stimulus, lack of time restriction from observation to response in Experiment 1 may have induced a longer gravitational simulation. Again, the work of De Sá Teixeira et al. (2013) supports this notion showing greater vertical displacement for longer response delays. Our findings are in line with this and other previous work on spatial displacement along with theoretical assumptions of simulating gravity. However, additional follow up work is needed to confidently back up the claim that differences in body orientation influence gravitational motion simulation. Controlling for the position of the man to imply various gravitational velocities while varying lag time between stimulus presentation and participant response could make for a more rigorous test of gravitational motion simulation in general, and in the case of object orientation or motion language specifically.

### SIMULATION ACCOUNTS OF MOTION FROM BODY ORIENTATION AND LANGUAGE

Experiments 1 and 2 support the idea that body orientation can influence the body’s remembered location in space. From this, the simulation of motion specific to the body may occur during image comprehension. The observed effects are consistent with the body’s possible motion, such that when oriented downward, it is possible to push off the cliff to a position farther away from the cliff than when oriented upward though both conditions in Experiment 2 were closer to the cliff than actual. This further suggests that the comprehension of where a person is may involve simulation specific to the person’s future actions. If so, it is no surprise that the location of the body is remembered differently, specific to its orientation. This extends our understanding of spatial memory to include processes involved in comprehending and predicting motion specific to bodies (Blake and Shiffrar, 2007).

Alternatively, the effects arising from the simulation of linguistic content did not appear to be present. A prominent theory in cognitive science posits that motion simulation is central to language comprehension (Glenberg and Kaschak, 2002; Matlock, 2004; Gallese and Lakoff, 2005; Barsalou, 2008; Bergen, 2012). From this, it follows that if motion language is simulated, effects should be observed in spatial memory. However, our current study fails to reliably determine how spatial memory, if at all, is affected by linguistic input, at least in the stimuli used in the current study. Future work is needed to fully assess how linguistic content might influence spatial memory.

### ALTERNATIVE ACCOUNTS

One possible critique might suggest the implied gravitational effects found in Experiment 2 were due to the presentation of language below the center of the presentation of the man on the following screen. Given our account of how the presentation of language may have influenced the remembered location of the man in Experiment 1, it is possible that the presentation of language, though on a different screen entirely, may have influenced the remembered location of the man in Experiment 2. However, given the implied gravitational effect found in Experiment 1, where language was presented at the top of the screen, the effects observed in Experiment 2 cannot be due to the presentation of language prior to and below the presentation of the stimulus.

Another possible account for the effects of implied gravity could be basic motor movements from participants actively moving their arms. Specifically, moving one’s arm to estimate a spatial location might influence spatial estimation. The use of a probe-judgment task throughout many studies (see Hubbard, 2005) actively avoids the possible effects of motor movements. This critique is only reasonable if the known starting position of the arm was always below the estimated location. Yet, even if the participant’s starting location were always below the image, motor movements alone cannot explain the observed orientation effects. Indeed the effect of orientation remains robust in the face of possible motor movements by participants.

Another possible issue is that the effect of body orientation may have occurred because different orientations lead the image of the man to be perceptually different *and nothing more.* In other words, that the image was presented differently *at all* may have been the reason for the observed significant differences—subtle circumstantial effects of the use of a single image. Effectively, any image presented in a different orientation may lead to this effect, e.g., a rock or table. If so, this would imply that the simulation of bodily motion does not occur, and that comprehension of the image does not rely on the simulation or prediction of its future motion, at least not in the specialized sense of agent-based action. Though plausible, it seems more plausible to suggest that if simulation does not occur, the image would not be experienced differently *at all*. Future studies will need to address the effects of object orientation that are specific to the properties of the object including agent-based action.

## SUMMARY

People are known to infer and anticipate motion in the world, including implied motion in static images. Yet there is still much to be discovered about how implied motion is realized, including how it varies across contexts, such as contexts that vary in terms of object position, implied gravitational forces, and linguistic information, if present. In the two experiments discussed in this article, we used an oﬄine task and an online task to show that spatial displacement occurs in the direction of implied gravity and obtained results that are consistent with previous implied motion work. We expanded the work on spatial memory by showing how influences from specific cognitive factors, such as body position, can potentially affect motion simulation. In doing so, we did not find an effect of language. Follow-up work on implied motion will further explore the role of language and agent motion on implied motion and spatial memory.

## Conflict of Interest Statement

The authors declare that the research was conducted in the absence of any commercial or financial relationships that could be construed as a potential conflict of interest.

## Footnotes

1 In an initial exploration (*N* = 302), the effects of Language were not consistent with Experiment 1. After a power analysis and an additional 299 subjects (*N* = 601), still no language effect was observed for any variables. Because crowdsourcing affords large volumes of easily accessible data, we collected data from an additional 407 participants.

2 Experiment 2 can be accessed here: www.davevinson.com/exp/repmom/1.html

3 It is possible the temporal distance between verb presentation and image presentation (1000 ms) may have eliminated any potential effects of language observed in experiment 1. The intent of this paper was in part to explore the possible constraints that may have stemmed from a survey-based experiment (e.g., presenting language with the image). Because of this, our study controlled for time in Experiment 2, though much data does suggest time between stimulus presentation and judgment influence spatial memory. For this reason, future studies may include the use of audio files to help control for the potential effect of stimulus onset asynchrony by allowing the presentation of language and image to occur simultaneously.
